# E- Learning experience of the medical profession’s college students during COVID-19 pandemic in Saudi Arabia

**DOI:** 10.1186/s12909-021-02860-z

**Published:** 2021-08-20

**Authors:** Eidan M. Al Zahrani, Yaser A. Al Naam, Saad M. AlRabeeah, Deemah N. Aldossary, Lamiaa H. Al-Jamea, Alexander Woodman, Mohammad Shawaheen, Osama Altiti, Jenifer V. Quiambao, Zechariah J. Arulanantham, Salah H. Elsafi

**Affiliations:** 1 College Deanship, Prince Sultan Military College of Health Sciences, Dhahran, Saudi Arabia; 2 Department of Clinical Laboratory Sciences, Prince Sultan Military College of Health Sciences, P.O. Box 33048, 31448 Dhahran, Saudi Arabia; 3 Department of Respiratory Care, Prince Sultan Military College of Health Sciences, Dhahran, Saudi Arabia; 4 Department of Anesthesia Technology, Prince Sultan Military College of Health Sciences, Dhahran, Saudi Arabia; 5 Vice Deanship of Postgraduate Studies and Research, Prince Sultan Military College of Health Sciences, Dhahran, Saudi Arabia; 6 E-Learning & Distance Education Unit, Prince Sultan Military College of Health Sciences, Dhahran, Saudi Arabia

## Abstract

**Background:**

Worldwide, most of educational institutions have moved to online electronic learning methods because of the COVID-19 pandemic. On March 8, 2020, the Saudi Ministry of Education announced remote learning for public and private schools and universities as a preventive and precautionary measure to curb the spread of the coronavirus. The objective of this study was to explore the e-learning experience of the students of the colleges of health sciences with regard to the technical preparedness, academic achievements, e-learning advantages and limitations. A well-structured and validated questionnaire on a five-point Likert scale and open-ended questions about their e-learning experience was distributed to a heterogeneous purposive sample of the health sciences students in Saudi Arabian universities.

**Results:**

Of the 1288 respondents, of various demographical features a relatively higher proportion of 58.2 % agreed that they had enough information about the online learning. However, the proportion who reported receiving adequate guidance, technical support, and having satisfactory hardware and internet access to online learning were 48.1 %, 42, and 35.4 %, respectively. Of all participants, 40.8 % agreed that they had gained a good understanding of their courses learning outcomes. Only 30.0 % agreed that the quality of the online teaching was similar to traditional classes and 56.1 % agreed that the online learning is unsuitable for the medical sciences studies.

E-learning advantages mentioned were the flexible accessibility of the learning materials, time, effort, and money saving, acquiring and improving technical and self-learning skills, health safety, interaction without shyness, and better academic accomplishment. On the other hand, disadvantages and difficulties included inadequate tools to facilitate online learning, poor internet connection, lack of technological skills by the educators and students. In addition, there was inadequate or lack of practical classes, lack of a unified clear policy for the conduct of online classes and exams and grade distribution, limited online exam time.

**Conclusions:**

The sudden shift to e-learning without prior preparedness has revealed some pitfalls that need to be adjusted. The initial findings were considered satisfactory for such a new experience for both learners and students. However, there is a great chance for improving and expanding the e-learning process.

## Introduction

The COVID-19 pandemic has enforced the closure of educational institutions worldwide. As a result, educational institutions have moved to online electronic learning methods, commonly referred to as e-learning, utilizing various hardware and software, as a possible substitute for the traditional face-to-face instruction. More than 107 countries had implemented a nationwide e-learning system by March 2020 [1]. Nevertheless, e-learning in higher education has progressively increased over the past two decades [2].

E-learning is a broad concept that involves the delivery of educational programs through electronic systems that rely on the internet for educator/student interaction and the dissemination of class materials [3, 4]. It involves the implementation of the advanced technology to plan, design and deliver the learning content, and to facilitate two-way communication between educators and students [5].

Several studies have demonstrated the usefulness of e-learning in higher education together with its limitations. E-learning is flexible in terms of time and place, easily accessible, and cost-effective [6, 7]. However, lack of direct communication, interaction, and instant feedback remained a distinct disadvantage. In addition, not all disciplines can effectively use e-learning in education such as health science studies that require hands-on practical experiences [8, 9]. Many studies compared e-learning with traditional face-to-face approaches. The effectiveness of e-learning is mostly determined based on how efficiently e-learning has been conducted compared to traditional full-time learning with the same content [10–12]. Moreover, some studies have considered e-learning as a potential tool in undergraduate medical teaching [13]. Thus, e-learning can be described as a tool that can make the teaching and learning process more student centered, innovative, and flexible. However, the major aspects of e-learning that have been consistently explored are its usefulness and the learner’s satisfaction. Several studies have shown that e-learning is mostly as good as traditional methods [14].

The expanding use of e-learning methods during the COVID-19 pandemic has necessitated the evaluation of their success in the teaching and learning processes. As a result, many institutions have become interested in how to deliver course content online effectively.

On March 8, 2020, the Saudi Ministry of Education announced remote learning for public and private schools and universities as a preventive and precautionary measure to curb the spread of the coronavirus. It is of great value to describe the first experience of the sudden shift to the e-learning system, thus forming a baseline data and providing data for possible improvements.

The current study’s objectives are to explore the e-learning experience of the students of the colleges of health sciences in Saudi Arabia with regard to the technical preparedness, academic achievements, e-learning advantages and limitations, and their recommendations for improving e-learning.

## Methods

 The Ethics Review Board of Prince Sultan Military College of Health Sciences, Dhahran approved this study (IRB Number IRB-2020-CLS-29). All methods were carried out in accordance with relevant guidelines and regulations. Every participant signed a written informed consent.

A well-structured, validated, and pretested questionnaire was designed according to the objectives of the study. The questionnaire was developed by the study group following a critical literature review. The questionnaire was anonymized and the participants did not have to mention any personal information. A brief description about the study’s objectives and the confidentiality of personal data was prepared which the participants were asked to sign as an agreement for their participation in the study.

The first part focused on demographic variables of the participants followed by 22 questions in five sections on a five point Likert scale. This was followed by seven questions concerning the technical preparedness and difficulties encountering by e-learning learning, eight questions on students’ learning experience, four questions on the interaction and feedback, and three questions on the student’s satisfaction with the e-learning courses assessment’s methods. The second part included open-ended questions in order to obtain a greater depth of information. The open-ended questions were about the advantage and disadvantage, the difficulties encountered, and suggestions for improving the e-learning process.

After completing the initial questionnaire items, two experts joined the panel to participate in reviewing the questionnaire items to establish face and content validity. After a series of meetings and adjustment to the questionnaire items, the members agreed on the survey sections and approved the final version. To test the instrument’s reliability, the final version of the questionnaire was distributed to a sample of the targeted population consisting of 78 students, who were not included in the study, to generate a Cronbach alpha value. The overall Cronbach’s alpha value for the survey items was 0.93.

The questionnaire was then distributed to the target population via a web link by utilizing various social media applications and was made available from 03 June to 23 December 2020.

A heterogeneous purposive sample of the health sciences students in Saudi Arabian universities who received an e-learning education during the COVID-19 pandemic were included in the study. All questions in the electronic questionnaire were identified as mandatory; therefore, it was necessary for the participants to choose one of the offered choices.

### Statistical Analysis

Statistical Package for Social Sciences (SPSS) 25.0 was used to analyze the data. Descriptive statistics were conducted to identify the level of agreement of the participants with the questionnaire items. The participant’s responses to the first part of the questionnaire were measured by questions on a five-point Likert scale rating, ranging from strongly agree (5), agree (4), neutral (3), disagree (2), and strongly disagree (1). The mean score of every question was calculated. The average scores on the technical preparedness for e-learning learning and difficulties were calculated out of 30 points for the six related questions. The average scores on the student’s learning experience were measured out of 45 points for the nine questions. The questions related to the student’s interaction and feedback during online courses were calculated out of 20 for the 4 related questions. Finally, average scores of the student’s satisfaction with the online courses assessment methods were calculated out of 15 for the three questions. The results were analyzed with the use of SPSS software version 20.0 (SPSS, Chicago, Illinois). The student’s response to the open end-questions were analyzed using the framework for thematic qualitative analysis as previously described [15]. One-way ANOVA was used to test the significant differences between the various e-learning parameters and the demographic variables. The statistical significance was set at P < 0.05 for all analyses.

## Results

Out of the 1367 respondents, the study included 1288 (94.2 %) who completed the questionnaire successfully. The participant’s demographical variables are shown in Table 1. The female participants comprised 63.7 % of the total. Those between the ages of 18 and 25 years constituted a larger proportion of 89.1 %. The vast majority of the respondents were Saudi citizen (93.8 %). Applied Health Science students were represented by 28.3 %, followed by Medicine (18.6 %) and nursing (16.9 %) students. The majority of the participating students were from the Central, Eastern, and Southern regions who represented 31.7 %, 28.8 %, and 21.9 %, respectively. The vast majority of the students were in bachelor programs (84.5 %). The various student’s academic levels were represented by 21.4 %, 20.7 %, and 21.3 % for year 2, 3, and 4, respectively.

**Table 1:** Demographic feature of the total participants (*n* = 1288)

Demographic factors	Frequency	Percentage
**Gender**		
Male	467	36.3
Female	821	63.7
**Age group**		
18 – 21	628	48.8
22 – 25	519	40.3
26 – 29	61	4.7
30 and above	80	6.2
**Nationality**		
Saudi	1204	93.5
Non-Saudi	84	6.5
**College**		
Medicine	240	18.6
Dentistry	163	12.7
Pharmacy	122	9.5
Nursing	218	16.9
Applied Health Science	365	28.3
Public Health	180	14.0
**Region**		
Central	404	31.7
Eastern	367	28.8
Western	166	13.0
Southern	279	21.9
North Western	7	0.5
North	52	4.1
**Study Degree**		
Diploma	64	5.0
Bachelor	1088	84.5
Master	33	2.6
Ph.D.	24	1.9
Other	79	6.1
**Academic Year**		
First year	167	13.0
Second year	276	21.4
Third year	266	20.7
Fourth year	274	21.3
Fifth year	176	13.7
Sixth year	82	6.4
Seventh year	47	3.6

Table 2 represented the participant’s response to the questionnaire. Of the all participants, 48.1 % agreed that they received an adequate guide to e-learning while 23.0 % did not agree. Similarly, 42.0 % agreed that technical support provided during the e-learning was adequate in contrast to a 32.5 % disagreement. A relatively higher proportion of the respondents of 58.2 % agreed that they had enough information about the e-learning platform (Blackboard Collaborate, ZOOM) while 21.0 % did not. Personal hardware and internet access were reported to be satisfactory for the learning process by 35.5 % in contrast to 44.1 % who disagreed. Technical difficulties during e-learning were encountered by 43.7 % while 32.5 % did not report it.

**Table. 2:** The participants rating of the various e-learning aspects as the total agreement (strongly agree and agree) or disagreement (strongly disagree and disagree) and the average score (out of 5) on the 5-point Likert scale (*n* = 1288)

E-learning experience	Agree	Disagree	Score out of 5
**Technical requirements**			
I received adequate guidance during the online learning.	48.1	23.0	3.35
Adequate technical support was provided.	42.0	32.5	3.12
I had enough information about the online learning platform.	58.2	21.0	3.53
I had no major technical difficulties during my online learning.	43.7	32.5	3.18
Online teaching materials were timely made available.	57.6	24.0	3.49
Online teaching materials were easily accessible.	46.3	26.0	3.45
My hardware and internet access were satisfactory.	35.4	44.1	3.12
**Learning experience**			
I gained a good understanding of the courses learning outcomes.	40.8	37.9	3.00
Overall, I had valuable learning experiences.	46.9	31.2	3.19
Virtual practical sessions were effective.	39.7	43.7	2.88
The quality of the online course was similar to traditional classes.	30.0	48.8	2.66
The online learning is suitable for the health professions studies.	24.4	56.1	2.42
**Interaction and feedback**			
I was able to interact with the instructor during the course.	49.9	29.9	3.29
Communication with my instructor was effective.	45.1	33.0	3.17
I was able to interact with other students during online learning.	57.3	24.0	3.48
The instructor provided me with feedback on my progress.	37.5	42.4	2.91
**Assessment methods**			
The examination process was clear to me.	52.1	28.8	3.35
The examination process was fair.	37.7	42.6	2.89
The overall assessment plan was satisfactory.	44.2	33.0	3.16
**Overall satisfaction**			
Online learning is stressful experience.	41.8	36.8	3.11
I was satisfied with the online learning experience.	41.5	35.7	3.05
I would like to study more online courses.	38.2	44.2	2.84

Of the total respondents, 57.6 % agreed that online teaching materials for all courses were made available in a timely manner during the study period, while 24 % did not agree. Easy accessibility of online teaching materials was described to be optimum by 46.3 % of the respondents unlike 26.0 % who did not agree with this.

Of all participants, 40.8 % agreed that they gained a good understanding of the course learning outcomes during online teaching while 37.9 % did not agree. In addition, 46.9 % agreed that they had valuable learning experiences during online teaching while 31.2 % did not agree.

About 39.7 % agreed that virtual practical sessions were effective during the e-learning while 43.7 % did not. Only 30.0 % agreed that the quality of the online teaching was similar to traditional classes while 48.8 % did not. Only 24.4 % agreed that the e-learning is suitable for the medical sciences studies versus 56.1 % who did not think so.

Ability to interact with the instructor during the course discussions was considered adequate by 49.8 % in contrast to 29.9 % who did not agree. Of the total participants, 45.1 % described their communication with their educators as effective while 33.0 % of them did not.

Interaction with other students during e-learning was described as adequate by 57.3 % unlike 24.0 % who were not able to do so. Only 37.5 % of the respondents agreed that the instructor provided them online with feedback on their work and progress whereas 42.4 % did not agree.

Of the total participants, 52.1 % agreed that the electronic examination process was clear to them while 28.8 % did not agree on this. Moreover, 37.7 % of the participants agreed that the electronic examination process was fair in contrast to 42.6 % of them who did not agree. Lastly, 44.2 % agreed that the overall assessment plan was satisfactory while 33.0 % did not.

Out of the total respondents, 41.5 % were satisfied with the overall e-learning experience versus 35.7 %. In addition, 38.2 % would like to study more online courses while 44.3 % would not. Finally, 41.8 % considered e-learning a stressful experience while 36.8 % did not.

Tables 3 and 4 summarizes the average scores of the question groups on the technical requirements for online courses 7 questions (out of 35), learning experience (8 questions out of 40), interaction and feedback 4 questions (out of 20), and assessment methods 3 questions (out of 15). The result indicated a significant difference in the various colleges regarding the technical preparedness for e-learning, with the dentistry college having the highest score followed by the health allied sciences and health colleges (*P* = 0.002). Another significant difference was in the technical preparedness among various respondent’s age groups, being highest among the older age group of ≥ 30 years (*p* = 0.025). The results also indicated a significant difference in the technical preparedness for online courses between the universities of various regions of the country, being highest in the Western region, followed by the Eastern and Central regions (*P* = 0.00). The tables also indicate the various scores for the learning experience according to the various demographical factors. Similarly, significant differences were detected between the age groups, Colleges, and regions. The score increased with age (*P* = 0.00) and is highest in the dentistry’s followed by the applied health sciences, and the public health colleges. The results also indicated significant differences in the scores related to the interaction and feedback during e-learning between the age groups, various colleges, and regions. The highest being among the older age groups, dentistry, public health, and applied health sciences (Table 3). Table 3 also indicated a similar significant difference regarding the student’s satisfaction towards the online assessment methods between the various demographical factors.

**Table. 3:** Mean scores of the technical (out of 30) and the learning experience (out of 40) of the online learning (*n* = 1288) and the 95 % confidence interval (CI)

Demographic feature	Technical requirements			Learning experience		
	Mean	95 % CI	P	Mean	95 % CI	*P*-value
**Gender**						
Male	19.83	19.24–20.43	0.24	23.41	19.24–20.43	0.40
Female	20.28	19.84–20.73		23.00	19.84–20.73	
Age (years)						
18–21	20.50	19.99-21.00	0.025	22.78	22.12–23.45	0.00
22–25	19.64	19.08–20.20		22.83	22.13–23.54	
26–29	18.90	17.06–20.74		24.52	22.15–26.90	
≥ 30	21.21	19.74–22.68		26.96	25.01–28.91	
**College**						
Medicine	19.82	19.04–20.61	0.002	21.85	20.73–22.98	0.00
Dentistry	21.87	20.87–22.87		26.09	24.81–27.36	
Pharmacy	19.80	18.54–21.05		23.16	21.63–24.68	
Nursing	19.06	18.14–19.98		22.48	21.31–23.64	
AHS	20.24	19.60-20.88		23.32	22.48–24.16	
HS	20.18	19.21–21.14		22.66	21.49–23.83	
**Region**						
Central	20.66	19.99–21.32	0.00	23.92	23.08–24.76	0.00
Eastern	20.77	20.12–21.41		24.28	23.44–25.12	
Western	21.04	20.14–21.94		22.10	20.85–23.34	
Southern	18.08	17.35–18.82		21.54	20.54–22.54	
N western	17.00	11.30–22.70		19.43	12.11–26.75	
North	19.98	18.04–21.92		22.35	19.83–24.86	
**Study Degree**						
Diploma	19.14	17.51–20.77	0.077	20.95	18.81–23.10	0.269
Bachelor	20.33	19.95–20.71		23.24	22.74–23.74	
Master	18.21	15.55–20.87		23.91	20.97–26.85	
PhD	18.42	14.89–21.94		22.21	17.56–26.85	
Other	19.27	17.73–20.81		23.58	21.50-23.61	
**Academic Year**						
First year	20.40	19.35–21.44	0.535	23.25	21.99–24.50	0.210
Second year	20.67	19.92–21.42		22.74	21.70-23.77	
Third year	19.93	19.09–20.77		22.88	21.84–23.92	
Fourth year	19.76	18.98–20.53		22.58	21.59–23.92	
Fifths year	20.03	19.13–20.94		23.90	22.68–25.13	
Sixth year	19.33	18.00-20.65		24.07	22.17–25.97	
Seventh year	20.77	18.81–22.71		25.57	23.10-28.05

**Table. 4:** Mean scores of the interaction and feedback (out of 20) and assessment method’s satisfaction (out of 15) of the online learning (*n* = 1288) and the 95 % confidence interval (CI)

Demographic feature	Interaction and feedback			Assessment methods		
	Mean	95 % CI	P	Mean	95 % CI	P-value
**Gender**						
Male	12.77	12.31–13.23	0.62	9.53	9.16–9.90	0.35
Female	12.91	12.58–13.23		9.32	9.06–9.58	
**Age (years)**						
18–21	12.88	12.51–13.25	0.009	9.49	9.19–9.79	0.010
22–25	12.54	12.12–12.96		9.07	8.73–9.41	
26–29	13.25	11.86–14.64		9.62	8.53–10.72	
≥ 30	14.46	13.42–15.51		10.56	9.73–11.39	
**College**						
Medicine	12.17	11.56–12.79	0.00	8.92	8.41–9.43	0.022
Dentistry	14.44	13.73–15.14		10.21	9.64–10.77	
Pharmacy	12.87	12.00-13.73		9.30	8.58–10.03	
Nursing	12.15	11.47–12.83		9.20	8.64–9.75	
AHS	12.92	12.43–13.40		9.61	9.32–9.99	
HS	13.07	12.37–13.76		9.14	8.57–9.72	
**Region**						
Central	13.40	12.91–13.90	0.00	9.72	9.35–10.09	0.019
Eastern	13.38	12.90-13.86		9.65	9.26–10.05	
Western	12.37	11.74–13.01		9.37	8.80–9.94	
Southern	11.85	11.28–12.43		8.69	8.22–9.17	
N Western	12.86	7.75–17.96		9.71	5.95–13.48	
North	12.19	10.77–13.62		9.33	8.10-10.56	
**Study Degree**						
Diploma	12.48	11.33–13.63	0.902	8.80	7.81–9.78	0.538
Bachelor	12.87	12.58–13.16		9.45	9.22–9.68	
Master	13.15	11.63–14.67		9.30	7.91–10.70	
PhD	12.21	9.85–14.57		8.46	6.42–10.50	
Other	13.04	11.92–14.15		9.44	8.54–10.35	
**Academic Year**						
First year	13.43	12.66–14.19	0.649	9.45	8.82–10.08	0.058
Second year	12.88	12.33–13.44		9.90	9.44–10.36	
Third year	12.51	11.91–13.12		9.20	8.73–9.66	
Fourth year	12.68	12.09–13.28		8.98	8.52–9.44	
Fifths year	12.97	12.30-13.63		9.14	8.57–9.72	
Sixth year	12.95	11.90–14.00		9.57	8.71–10.44	
Seventh year	13.09	11.68–14.49		10.34	9.29–11.39	

**Fig. 1:**
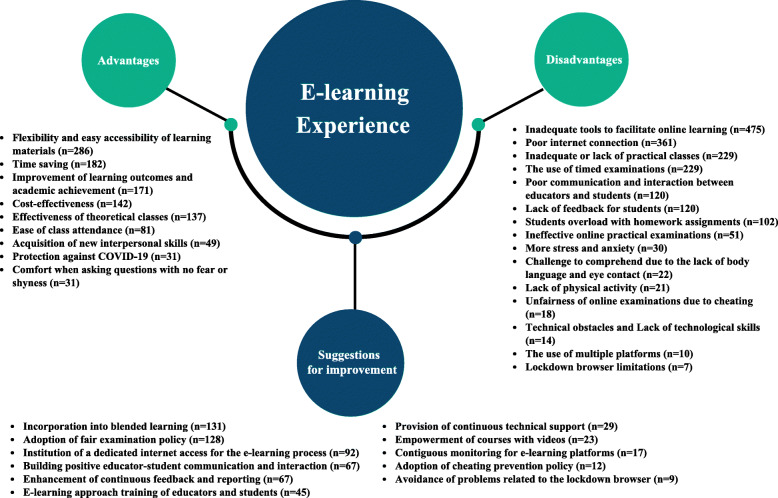
The frequencies of the advantages, disadvantages, and suggestions for improving the e-learning system provided by the participants (*n* = 1288)

The student’s opinions about the advantage, disadvantages and suggestions for improving the e-learning process are summarized in Fig. 1.

## Discussion

We investigated the various aspects of the e-learning experience by the students of medical science colleges in Saudi Arabia during the Covid-19 pandemic as regard to the technical preparedness, teaching and learning experience, interaction and communication, and assessment methods. The study demonstrated that a relatively higher proportion of the respondents 58.2 % agreed that they had enough information about the e-learning platform. However, less than 50 % of the total participants agreed that they received adequate guidance and technical support during the e-learning process. It is well known that the success of the e-learning program depends largely on the learning tools and the technical support available for the users [16].

The e-learning standards for Higher Education in the Kingdom of Saudi Arabia were built according to the best-known international standards that include technology, training and support, design, interaction, equity and accessibility, and assessment and evaluation [17]. Every e-learning system establishes its basic foundation of computers, networks, communications and technical facilities along with information communication technology professionals to continuously maintain and upgrade the system, train the users and provide technical support [18]. Appropriate technological support and maintenance of the available hardware and software is of great value for utilizing the technology by educators and students up to the optimum [19–21]. Personal hardware and internet access in the current study were reported to be satisfactory for the learning process by 35.5 % in contrast to 44.1 % who thought them to be unsatisfactory. A larger proportion of the study’s respondents agreed that easily accessible online teaching materials for all courses were made available in a timely manner during the study period. It has been stated that the success of the online education program is related to the provision of adequate levels of educational guidance and technical support [22, 23].

In this study, we noticed a significant variation in the fulfillment of technical requirement of the e-learning process within the various colleges and regions. Dentistry, applied health sciences, and public health colleges were the best prepared for e-learning because of their previous experience prior to the COVID-19 pandemic [24]. The variation between regions is proportional to the socioeconomic parameters of the different regions [25].

Evidences for the equivalency of e-learning to traditional methods with respect to the learners’ achievements in knowledge widely exist. However, the current findings do not fully support this, since nearly half of the respondents did not agree on the similarity of e-learning and traditional classes in terms of achievements of learning outcomes. Most of them mentioned the unsuitability of e-learning for the health sciences studies. In addition, a significant difference in the learning experience was detected between age groups, colleges, and region. Older age group of ≥ 30 who are mature students and enthusiastic younger ones of 18–21 years who are in the first years reported the best valuable learning experience.

e-learning modalities that are flexible and effective sources of teaching and learning cannot be free of some adverse circumstances. According to the educators and students, e-learning is a flexible and effective source of teaching and learning that helps in distance education with less use of resources and time. The flexibility of e-learning over face-to-face teaching has been reported in the literature previously. This makes the students to become self-directed learners, an important competency for supporting lifelong learning among health care professionals [26, 27].

The majority of our respondents considered that e-learning is unsuitable for the health sciences studies. Several meta-analysis studies have demonstrated that learning at a distance in health-allied sciences is as effective as traditional classroom instruction [28, 29]. However, some barriers or challenges exist. Faculty members and students said that through e-learning modalities, practical and clinical work are not properly taught and learned.

Barriers and challenges of e-leaning modalities are many. One of these is poor motivation and an expectation to be able to meet their personal and professional needs and goals [30].

Internal factors such as poor engagement, poor perception and motivation, high levels of anxiety and stress, and poor interactions between learners and facilitators hinder the process of learning and motivation [31, 32].

Most of the participating students in the current study admitted that they gained a good understanding of the courses learning outcomes during the e-learning experience. This was considered to have been a valuable outcome. It has been stated that well-designed virtual learning may result in more effective learning in comparison with the traditional face-to-face training [33]. The majority of our respondents did not agree on the similarity of e-learning and traditional classes. Many studies reported no significant difference between the two teaching methods. Overall, it has been suggested that e-learning is at least as effective as traditional face-to-face learning. However, others showed that e-learning was better than offline learning [34, 35].

In this study, the majority of students disagreed on the effectiveness of the virtual practical sessions as a replacement for real laboratory training. It has been recommended that a blended teaching strategy that includes in-person laboratory training is more appropriate.

Virtual laboratories, and video-based laboratories are better choices when students are not physically located on campus [36]. It has been stated that online teaching and laboratory practices in the biosciences field is often more effective than traditional based learning [37].

A large proportion of the participants of the current studies have considered e-learning as a stressful experience. In a cross-sectional study among Saudi Arabian medical students, pandemic-related anxiety and stress were specified among the challenges [38]. Most of the participating students in this study stated that they would not like to take more e-learning more classes if they were given the option. Stress has been many times associated with e-learning than with traditional learning [39, 40]. Increased concerns in academic performance as a stressor contributing to increased levels of stress, anxiety, and depressive thoughts among students due to the COVID-19 pandemic situation was previously identified [41].

 A large proportion of our participants admitted their ability to interact effectively with the instructor and even more of them were able to interact with other students during the e-learning classes. There has been a great concern that the physical interaction during online courses is not up to the standard which might affect the learning outcomes [42]. However, meaningful interaction between educators and students can be achieved in online courses through discussion forums [42]. Encouragement of interaction during the online classroom is an important thought to ensure the active knowledge [43]. While students indicated that the interactive components of the course were valuable, several areas in which improvement may be made remain, such as lack of participation on the part of fellow students in discussion and inability to attend synchronous sessions due to course scheduling conflicts [43].

Several studies have provided evidences for the relationship between student-student interaction, student-educator interaction and academic emotions and learning persistence [44, 45]. It has been proposed that student-student discussions could boost higher levels of knowledge construction and learning outcomes in student-student discussions [23].

Our findings detected a significant difference in the interaction and communication during the e-learning experience between age groups, colleges, and regions. Again, the older age group of ≥ 30 and younger ones of 18 -21years reported their ability to better interact between educators and among themselves during the online classes. Dentistry, Applied Health Science and Health Sciences College have reported better interaction and communication than their peers in other colleges. Similarly, the central and Eastern regions have shown better interaction.

An appreciable number of the participants (42.4 %) regarded their instructor’s feedback on their work and progress as poor. Feedback is an important component of effective learnerning [46]. Feedback provision supports the opportunity for enhanced academic performance. Meaningful feedback on assignments enhances critical thinking, reflective practice, and develops educator-student relationships which is important in an e-learning process.

Another adequate number of 44.2 % considered the overall assessment plan as satisfactory. Although many considered that the examination process was clear to them, a large number regarded the online examination process as unfair, mostly due to the intentional short duration of the examination period. Student satisfaction is important because it predicts student retention and is linked to the student’s learning outcomes [47]. Varied results have been reported when comparing student satisfaction in face-to-face and online courses [48].

Part of the study included open-end questions to obtain detailed responses and real insights. Our respondents mentioned the flexible accessibility of the learning materials, time, effort, and money saving, acquiring and improving technical and self-learning skills: Self-learning / Improvement in technology skills, safety from COVID-19, good interaction between the instructor and students with no shyness, and better academic accomplishment. On the other hand, disadvantages and difficulties included inadequate tools to facilitate e-learning, poor internet connection, lack of technological skills by the educators and students. Also there was inadequate or lack of practical classes, lack of a unified clear policy for the conduct of online classes and exams and grade distribution, limited online exam time. Other difficulties mentioned included lack of body language and eye contact and direct communication and active discussion. Additionally there were distractions, lack of motivation and anxiety, ineffectiveness of online practical, and unfairness of assessment methods due to cheating.

Several suggestions were mentioned for improving the e-learning process. These included the use of blended learning strategies that include an in person practical/clinical training. Other suggestions included staff and student’s technical training, more use of visual training materials, more time for exams with multiple attempts, the existence of examination policy during internet disconnection, the use of an effective system that detects and prevent cheating, and encouraging active interaction between the instructor and students. To avoid the potential limitations of e-learning in undergraduate medical education, it might be worthwhile to combine the advantages of online and offline teaching methods, called blended learning [49]. However, there is still a need for more scientific evidences for clear comparison between online and traditional learning, since most experimental designs of the previous research articles varied in terms of participants, learning goals, intervention durations, and forms of e-learning, etc. It has been suggested before that the low use of social networking sites for sharing information along with the great variations in the medical student’s perceptions about social media should draw attention of the concerned institutions to advancing awareness and educational transforms [50].

The main limitations of the study is the cross sectional design and the small sample size. Another major limitations of our study was the possibility of recall bias of our respondents.

## Conclusions

The sudden shift to e-learning without prior preparedness has revealed some pitfalls that need to be adjusted. The initial findings were considered satisfactory for such a new experience for both learners and students. However, there is a great chance for improving and expanding the e-learning process.

## Data Availability

Data collected during this study is safely protected in Google drive. The datasets used and/or analysed during the current study are available from the corresponding author on reasonable request.
